# Early complications of percutaneous K-wire fixation in pediatric distal radius fractures—a prospective cohort study

**DOI:** 10.1007/s00402-023-04996-7

**Published:** 2023-07-31

**Authors:** Michał Wasiak, Maciej Piekut, Karol Ratajczak, Marcin Waśko

**Affiliations:** 1 Department of Orthopedics and Traumatology, Medical University of Warsaw, Warsaw, Poland; 2 Department Orthopaedic Surgery and Traumatology, Popiełuszko Memorial Bielański Hospital, Warsaw, Poland; 3 Department Orthopaedic Surgery and Traumatology, Bogdanowicz Memorial Hospital, Warsaw, Poland; 4 Department of Radiology and Imaging, Centre of Postgraduate Medical Education, Warsaw, Poland

**Keywords:** Pediatric distal radius fracture, Complication, Percutaneous pin fixation, Infection

## Abstract

**Introduction:**

Distal radius fractures (DRF) are the most common pediatric fractures, but the current evidence for management remains inconclusive. Closed reduction and percutaneous pinning (CRPP) provide excellent stability but are not complications-free. Therefore, a thorough evaluation of their adverse events is necessary to provide reliable information on risks and benefits in different clinical scenarios. The current literature lacks studies conducted with rigorous grading systems and uniform follow-up protocols on this topic. This prospective cohort study used a validated grading scheme to analyze complications associated with CRPP in an unselected pediatric population with displaced, unstable distal third radius fractures.

**Materials and methods:**

One hundred and nineteen DRFs (one hundred and sixteen patients) treated with CRPP were enrolled in the study. All patients were followed 4 weeks, 5 weeks, 3 months, and 6 months after the surgery. The same protocol, comprising structured history, physical and radiological assessment, was used throughout the study. All data were prospectively abstracted. The Clavien–Dindo–Sink grading system was used to assess the complications and the Dahl score to evaluate the pin sites.

**Results:**

Forty-two wrists (35,3%) had CDS grade I or II complications, and two (1,7%) had a grade III complication. The general complication rate for the study group was 37% (44 complications). Two patients required repeated surgery—deep bone pin-track infection treated with the Masquelet technique and surgical removal of a migrated pin. Among minor complications, pin-site inflammations were the most common—40 wrists (33,6%).

**Conclusions:**

The CRPP is a safe treatment method for DRF in pediatric patients, with a low major complication rate. However, minor adverse events are frequent and can significantly burden the patient’s postoperative well-being. The application of rigorous definitions and grading systems should not only lead to the obtainment of high-quality data but also to higher awareness of possible pin tract infections and therefore allow for better therapeutic decisions.

## Introduction

Distal radius fractures (DRF) are the most common pediatric fractures [1]. Closed, reducible fractures without neurovascular compromise are treated with either cast immobilization or manipulation under anesthesia, followed by cast immobilization [2, 3]. For patients with severely displaced fractures, when reduction cannot be maintained by plaster cast or secondary displacement is expected, closed reduction followed by percutaneous pinning (CRPP) is considered a viable treatment [2, 4–6]. However, the evidence for management remains inconclusive, and decision-making can be challenging for a physician [2, 4, 6].

Probable treatment complications remain an essential issue that must be considered when choosing an optimal clinical pathway. The complication rates reported for the CRPP in DRF vary from 0 to 38% [4, 6–18]. This wide variation stems from the different methodologies used, e.g., skin redness can be classified as an infection [19]. However, regardless of the methodology, most complications can be attributed to the presence of the Kirschner wires [4, 5, 7, 14, 20]. With an increasing trend toward surgical fixation, systematic and thorough knowledge of CRPP complication rates and their pattern might help orthopedic surgeons better counsel their patients and their parents [21, 22].

This prospective cohort study used a validated grading scheme to analyze complications associated with CRPP in an unselected pediatric population with displaced, unstable distal third radius fractures.

## Materials and methods

### Study population

**Fig. 1:**
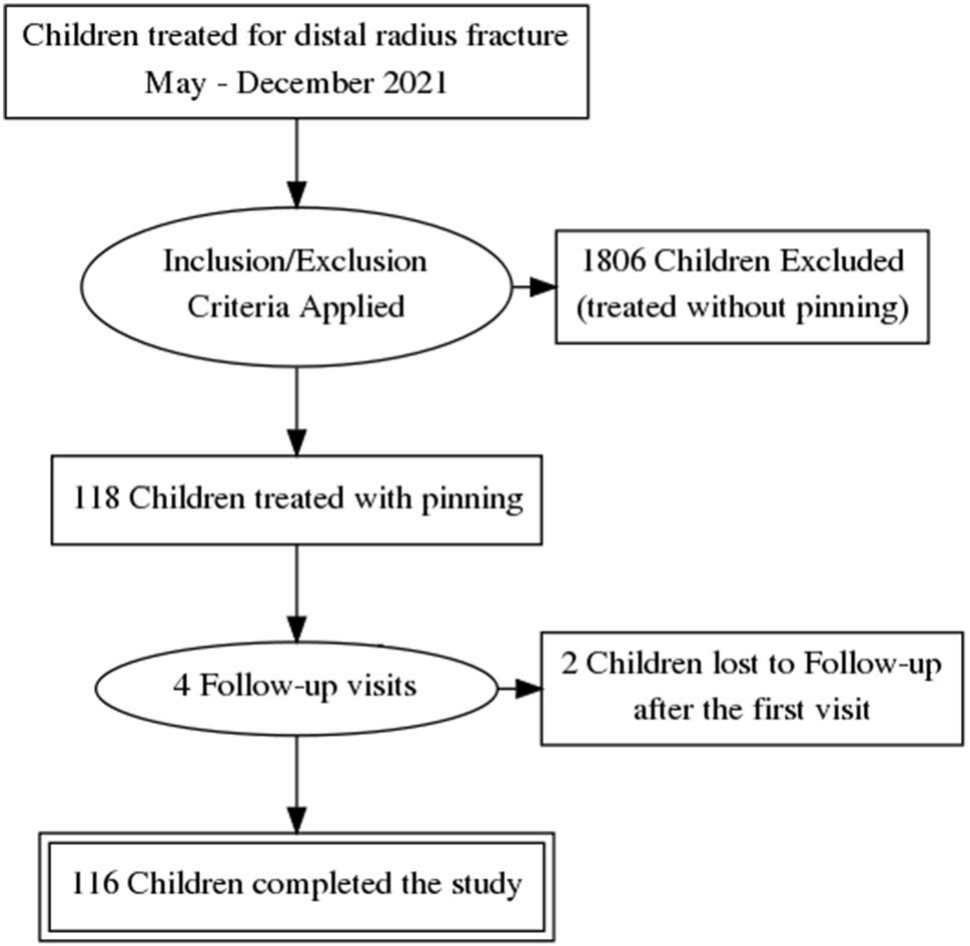
Study flowchart

The study was conducted in a 250-bed tertiary healthcare center. All consecutively hospitalized minor patients undergoing CRPP for DRF between May and December 2021 were enrolled in the study. The inclusion criteria were displaced unstable distal third radius fractures. The exclusion criteria from the study comprised pre-existing skin lesions, conservative treatment, and open fractures. During the study time, 1924 children with distal third radius fractures were treated at our institution. and 118 of them received closed reduction and percutaneous fixation. All patients were referred for follow-up in the hospital’s outpatient clinic. Unfortunately, two patients were lost to follow-up after the first visit (Fig. 1).

Therefore, one hundred sixteen patients – 75 boys (65%) and 41 (35%) girls finished a complete follow-up and were included in the final analysis (see Table 1). Age ranged from 3 to 15 years, with an overall median 11 (IQR: 9–13). The median of age for girls was 10 (IQR: 7–11) and median of age for boys was 12 (IQR: 9–13). Two patients had closed epiphyseal plates. The age difference was statistically significant (*p* = 0,001). Three patients (2,6%) had bilateral DRF treated with CRPP, and each side was included in the analysis as an independent event. Hence, the total number of analyzed fractures was 119.

**Table 1:** Study group characteristics

Characteristic	Number (percentage)
Sex	
Male	77 (66,4%)
Female	39 (33,6%)
Side of the fracture	
Right	41 (34%)
Left	78 (66%)
Salter-Harris fracture type	
Not a physeal fracture	73
I	2
II	43
IV	1
Pin placement pattern	
de Palma	52 (43,7%)
Kapanji	23 (19,3%)
intra-medullary	9 (7,6%)
intra-medullary single pin	35 (29,4%)
Concomitant distal ulna fracture	
Yes	63 (53%)
No	56 (47%)
Distal ulna CRPP	
Yes	6 (5%)
No	113 (95,5%)

*CRPP* closed reduction and percutaneous pinning

The children’s guardians provided written consent for the treatment. The local ethics committee approved the research project, classified this study as a quality control study, and waived the need to obtain additional informed consent (no. 1/2021). The reporting of this study conforms to the STROBE statement [23].

### Operative procedures

The patients were operated on within 12 h of the fracture. Before surgery, each patient received the first dose of the antibiotic prophylaxis per local guidelines (three doses of cefazolin every 8 hours, dosing for patients with a body weight of fewer than 25 kg – 3 mg/kg; for patients between 25 and 49 kg – 1 g, for patients over 50 kg – 2 g); two remaining doses were administered after the surgery. General anesthesia was applied. The operative field was prepped with a mixture of propanol and diphenylol (Kodan Tinktur Forte, Schülke & Mayr GmbH, Norderstedt, Germany). After obtaining closed reduction, 2.0 mm K-wires were inserted under fluoroscopy control. The pin tips were left at least one centimeter above the skin and protected with sterile gauze dressings. The mineral long arm cast was applied directly after surgery, covering the pin tips. The patient was observed in a surgical ward for a minimum of 12 h and discharged home, provided no complications emerged. The patients were referred for follow-up at the same institution.

### Standardized follow-up procedure

#### Follow-up schedule

The regular follow-up visits took place 4 weeks, 5 weeks, 3 months, and 6 months after the surgery. The follow-up protocol, adopted as routine conduct in the hospital, included standardized history, physical, and radiological examination and was used for all the visits, including additional visits for complications treatment (Table 2). Wire removal was performed during the first visit and cast removal during the second visit unless findings in physical or radiological examinations indicated contradictions. The dressing was left from the time of operation untill wire removal, unless any symptoms were reported by the patients or the guardians.

**Table 2:** Follow-up visit examination protocol

1. Interview
Problems tolerating the therapy—descriptive
Problems with a return to daily living activities—descriptive
Other complaints
2. Physical examination
Neurovascular assessment
Pin site inspection
The limitation of movement of neighboring joints
The limitation of movement of the afflicted joint
3 Radiological examination
Bony union and remodeling
Secondary displacement
Wire migration
Osteitis (local osteopenia, focal cortical or trabecular lysis, elevation of periosteum)
Other findings

#### History

The interview had a uniform structure. First, the patient and their guardian were asked to assess the child’s pain while resting according to the Numeric Pain Rating System (NRS) [24]. Next, any problems with the tolerance of the therapy or complaints were noted.

#### Physical examination

Physical examination during each visit consisted of: neurovascular assessment, pin site inspection, and range of motion assessment for the operated and contralateral limb [25]. In addition, the pin sites were carefully examined, paying particular attention to any signs of infection, inflammation, pressure ulcers, and pin migration.

#### Radiological assessment

Anteroposterior and lateral wrist views were obtained during each visit and assessed by musculoskeletal radiologists. The following radiological features were assessed: displacement, wire migration, osteitis (focal cortical or trabecular lysis, elevation of periosteum), bony union, and remodeling [26].

#### Assessing the complications

The overall postoperative course was assessed according to Clavien-Dindo-Sink (CDS) grading system [27]. The CDS grading system was validated for assessing surgical procedure complications, focusing on their management and impact on the patient’s health. Grade I represents a typical postoperative course with minor complications requiring no treatment and having no clinical relevance. Grade II comprises minor complications managed in the outpatient clinic. Complications of grade III require invasive intervention, whereas grade IV can be life-threatening and cause long-term disability. Finally, the grade V complication is death [27]. Separately, pin-site infections were classified according to Dahl. Grade 0 means a normal condition of a pin site; grade 1 indicates inflammation, grade 2 indicates serous discharge, grade 3 indicates purulent discharge, grade 4 indicates osteolysis, and grade 5 ring sequestrum in bone [28]. Other complications were noted descriptively. Physicians abstracted the postoperative course of each patient, noted complications by name and date of occurrence, and assigned appropriate grading. Differences in grading were resolved by consensus, inviting an independent attending surgeon as a third rater.

### Data collection

All patients’ medical records were abstracted prospectively and anonymized. The following data were abstracted and stored in an Excel spreadsheet for the study purpose: sex, date of birth, date of fracture and date of surgery, surgical details, pertinent previous medical history, presence, and type or absence of pin site complication, additional treatment, the final result of the treatment, and radiological outcomes.

### Statistical methods

In the case of normal distribution and equal variances, independent samples t-test was used to compare the means. In case of unequal variances, the Welch test was used. The chi-square test for categorical variables evaluated differences between groups as appropriate. All statistical analyses were performed using MedCalc version 18.5 software (MedCalc bvba, Ostend, Belgium), and significance was determined using an alpha level of 0,05.

### Source of funding

This research received no specific grant from any funding agency.

## Results

**Fig. 2:**
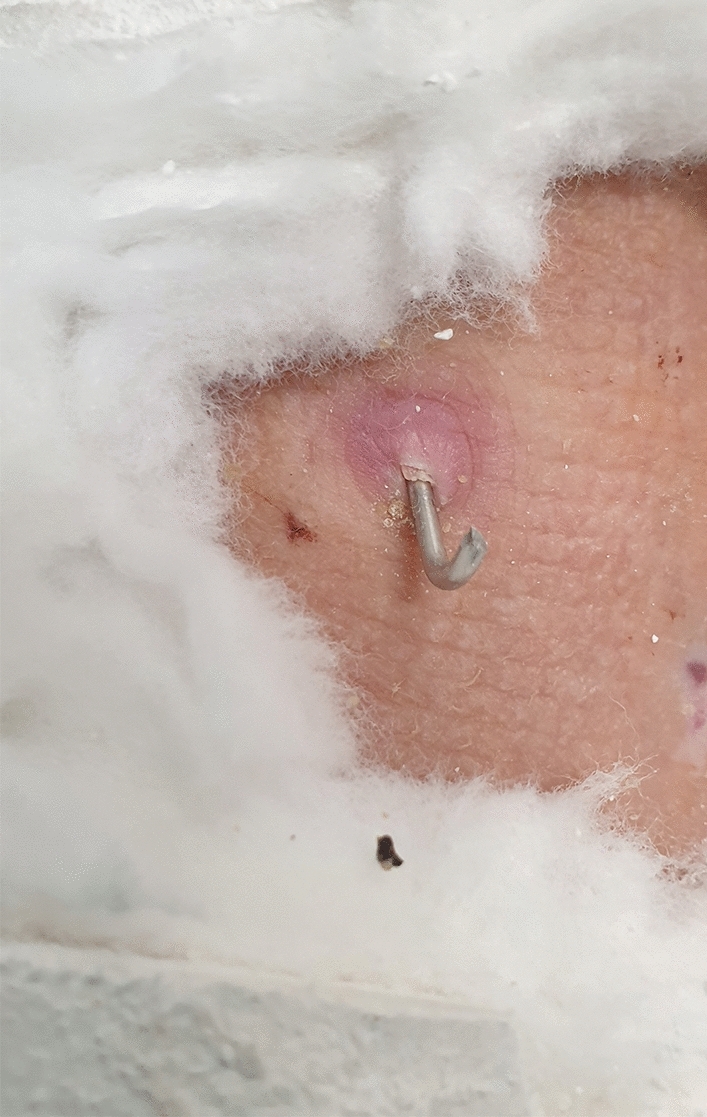
Mild inflammation around K-wire (Dahl grade 1)

**Fig. 3:**
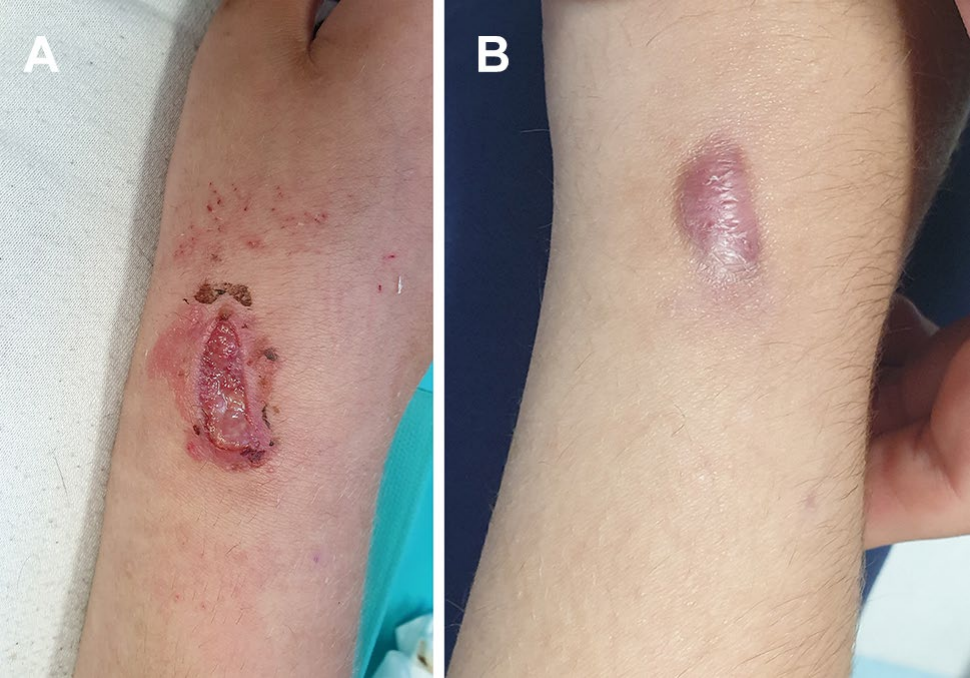
Ulceration of the pin-site just after wire removal (**A**) (Dahl Grade 3) and healed with prominent scar (**B**)

Of the 119 wrists (in 116 patients), 42 (35,3%) had CDS grade I or II complications (Fig. 2), and 2 (1,7%) had a grade III complication (Fig. 3A, B). No grade IV or V complications were noted. The general complication rate for the study group was 37% (44 complications – see Table 3). The most common complications were those related to the pin-site – 40 wrists (33,6%) (38 minor and 2 major complications).

**Table 3:** Complications summary

	Number (percentage)
The overall	44 (37%)
Pin-site	40 (33,6%)
Dahl Pin-Site Complication	
Frist visit	
Grade 1	26 (21,8%)
Grade 2	9 (7,6%)
Grade 3	2 (1,7%)
Grade 4	0
Grade 5	0
Second visit	
Grade 1	15 (12,7%)
Grade 2	1 (0,8%)
Grade 3	0
Grade 4	1 (0,8%)
Grade 5	0
Third visit	
Grade 1	0
Grade 2	0
Grade 3	0
Grade 4	0
Grade 5	0
Pressure ulcers	30 (25,2%)
Non-pin site	7 (5,9%)
Transient neuropraxia	1 (0,8%)
Limited range of movement	6 (5%)

**Fig. 4:**
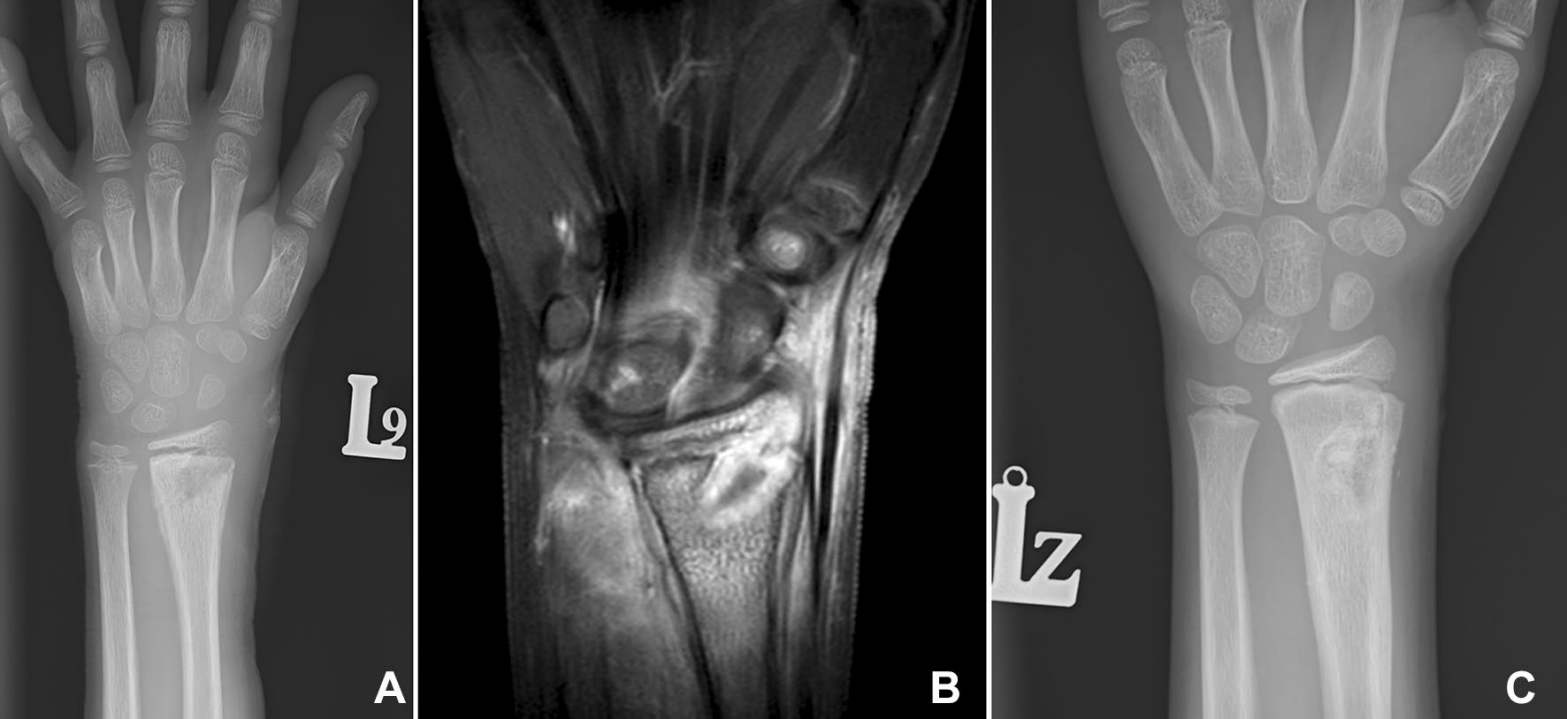
Bone osteolysis along the pin track (Dahl grade 4) X-ray (**A**), magnetic resonance (**B**) and X-ray after successful Masquelet procedure (**C**)

There were two significant complications requiring repeated surgery. First, a 6-year-old girl experienced wire migration beneath the skin, requiring surgical removal under general anesthesia. After removal, she healed uneventfully. The second major complication occurred in a 9-year-old boy who developed radius osteomyelitis. There was no discharge from the pin site between the first and the second visit. However, there was a pressure sore, which failed to heal within a week of local treatment. The X-ray and magnetic resonance confirmed the suspicion of deep infection of the distal radius (Fig. 4A, B). The patient underwent two-step revision surgery with a modified Masquelet technique [29]. First, debridement and gentamycin- and vancomycin-loaded bone cement spacer implantation were performed. Microbiological examination of samples taken during surgery revealed methicillin-sensitive *Staphylococcus aureus*. For four weeks of the postoperative period, the patient received cefuroxime axetil 250 mg two times per day, per the antibiogram. Within six weeks of the first surgery, the patient underwent the second step—bioactive glass (GlassBone Granules, Noraker, Lyon, France) was implanted following cement removal. The oral antibiotic was maintained for two weeks after the second stage and then withdrawn. The wound healed properly within 2 weeks of the second step. Nine months later, we noted no relapses of infection and the X-ray confirmed bone remmodeling (Fig. 4C).

**Fig. 5:**
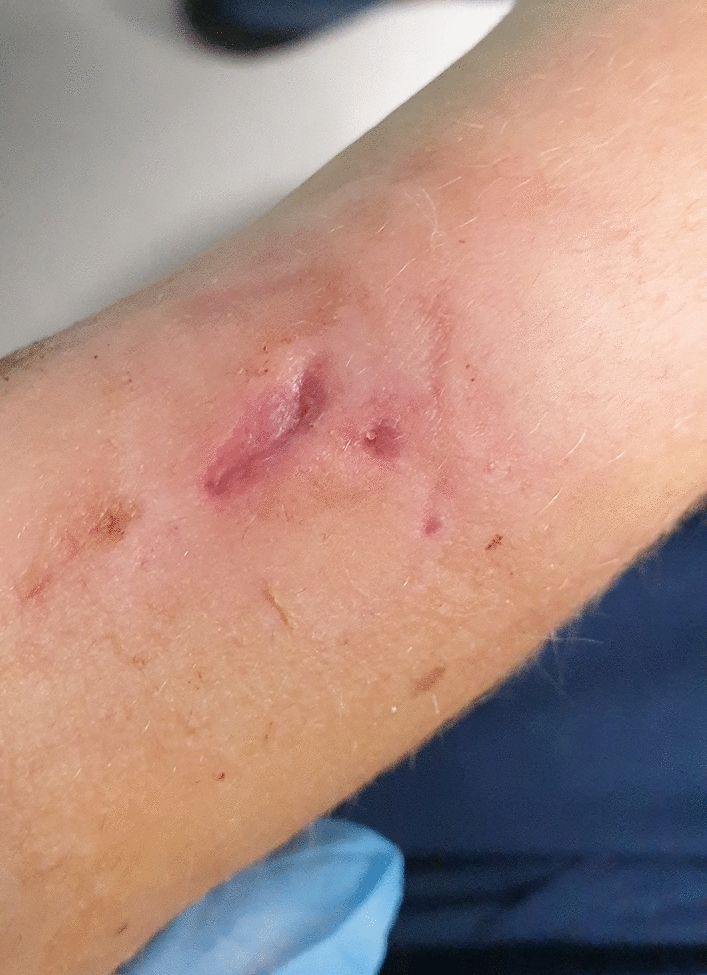
Pressure ulcer along the removed pin (Dahl grade 1)

The most common minor complication (CDS grade I or II) was pin-site inflammation. Such complications occurred in 38 wrists (31,9%), with pressure ulcers as the most common presentation (30 wrists – 25,2%) (Fig. 5). All those complications have been resolved without additional surgical intervention requiring only minor changes in pin site care (e.g., single silver-coated dressing application). Complete resolution occurred in all cases.

The complications unrelated to the pin-site included one case of transient ulnar neuropraxia, which resolved spontaneously within 5 weeks, and six cases of a limited range of motion, which resolved with proper rehabilitation.

There were no cases of re-displacement. We noted only a single case of ulnar styloid non-union, which did not require additional follow-up visits. We also noted two hypertrophic scars (1,7%). Those events did not trigger any change in the follow-up schedule, so we did not classify these three events as complications.

The patients treated with only one wire had significantly more complications than patients treated with two wires (*p* = 0,011). There was no significant correlation between complication rate, and gender (*p* = 0,899), age (*p* = 0,203) and pin placement pattern (*p* = 0,109).

## Discussion

The current evidence for managing distal radius fractures remains inconclusive [2, 4, 6, 30]. While further evidence accumulates, other criteria, such as the complication risk and the nature of the complications, may help guide treatment choice and patient and parent counseling. To our knowledge, the complications and their rate following CRPP for pediatric displaced unstable distal radius fractures have not been prospectively described with the use of rigorous grading systems and uniform follow-up protocols.

The reported overall complication rate varies from 0 to 38% [4, 6–18]. In two separate meta-analyses, the average was 28% [31] and 15,7% [32]. Following a strict protocol and a validated grading score, we identified complications in 37% of cases in our study. When comparing this data, one should consider that mentioned studies hold several limitations, including a low number of participants, lack of standardization in follow-up protocols, surgical techniques, and grading systems used to describe complications [19, 31, 32].

CRPP has been proven to significantly improve stabilization and reduce the re-displacement rate compared to reduction followed by casting [6, 14, 17, 32, 33]. Accordingly, we have not noted any cases of the loss of reduction, which remains consistent with other reports, indicating a low risk of re-displacement after CRPP [6, 8, 10, 11, 13] with an average of 3,8% in a single meta-analysis [32]. However, the use of pins leads to unique complications. In our group, 33,6% of pin-sites had an inflammatory complication, most commonly presenting as pressure ulcers, which were observed in 30 cases (25,2%). Except for one, all inflammatory reactions were limited to soft tissues, not involving bone, and were successfully managed by the 5 week postoperatively. In the literature, there is a wide range of reported prevalence of such complications. Passiatore et al. reported 9,6% ulcers among the patients treated with the EpiBloc system [16]⁠, while Miller et al. reported 38% of pin-related complications [4]⁠. Thus, most of the reported grade I complications, which constituted the majority of the overall complication rate, had little direct clinical importance. Nevertheless, even a minor complications can cause discomfort for the Patient. Hence, it is also important to investigate for a reason for its prevalence and ways to prevent them. According to the literature review conducted by Kazmers et. al, there is limited evidence on how to prevent pin site infection [19]. However, considering the fact, that most of the pin-site complications in our study were pressure ulcers, we believe that insufficient bending of the wires might have been the most significant contributing factor..

Unfortunately, one case of deep infection with bone osteolysis (grade 3 according to Dahl) was diagnosed in our study population. Such deep infections after DRF CRPP are rarely reported [5, 12, 34]. Even though our patient responded well to the treatment, he had to undergo two separate surgeries.

In two cases (1,7%), pins migrated beneath the skin and required removal – one of them (0,8%) in general anesthesia. This finding remains consistent with the low numbers reported by other authors, 0–11,6% [6, 11, 15, 17].

The patients treated with only one wire had significantly more complications than patients treated with two wires. This finding has not been described in the available literature. On the one hand, an increased complication rate may represent increased forces unloading at the single pin-skin interface. At the same time, it can also be related to different cast molding in the presence of only a single K-wire compared to casts overlaying two K-wires.

Because of the limited follow-up of the study time, we were not assessing long-term functional outcomes. However, all of our patients regained their full range of movement, which remains consistent with the available literature data. Limited motion is reported in 1,5–13% of patients and, in most cases, resolves over time [11, 13, 31].

Although this current study represents an important step in improving the evidence base and better-informing surgeons', parents’, and patients’ choices, it holds several limitations.

Firstly, even though the study is prospective, it lacks randomization in its part regarding the influence of age, gender, and pin pattern on the complication rate. However, broad, clinically-relevant inclusion provides a relatively unselected sample of patients presenting at the hospital and allows observing natural clinical patterns. Secondly, the results cover a minimal follow-up of 6 months, excluding complications like growth arrest or post-traumatic arthrosis from our analysis. However, our study focused on complications related to the surgical procedure of CRPP, and most of such adverse events appear shortly after surgery [4, 6, 17]. Third, we have not evaluated the impact of season-related atmospheric factors, which could deteriorate healing beneath the cast. Interestingly, such analysis was conducted for supracondylar humeral fractures and found a significantly higher infection rate in the high-temperature season [35]. Fourth, five surgeons performed CRPP, and differences between their operating techniques were not directly addressed in this study. However, all of them followed the same protocol for DRF management. The uniform treatment algorithm provided comparable timing of pin placement after trauma and removal after surgery and the use of the same diameter K-wires. In addition, we studied complications in a busy tertiary-care center in patients operated on by high-volume pediatric orthopedic surgeons. The complication rate might not be generalizable for surgeons with different practice profiles, training, and experience. Fifth, we did not include patient-reported outcome measures. However, we used the Clavien-Dindo-Sink classification to provide a subjective method of assessing complications. This classification exhibits excellent reliability in pediatric orthopedics, is widely used, and has been thoroughly validated [27, 36]. Lastly, we did not evaluate the costs of the therapy, which may limit the direct clinical application of our findings.

Using a validated grading scheme, we showed that CRPP remains a relatively safe treatment modality for pediatric patients with displaced, unstable distal radius fractures. Major complications occurred in less than two percent of the population. However, 35,3% of the patients experienced minor complications, which remains unacceptably high. Although these complications were easy to manage and did not lead to permanent disability, they unnecessarily added to patients' and parents' discomfort. The solution might lie in both physicians' education and the development of better wound care for the pin sites. This study outcomes also stress the need to develop precise criteria, indicating when the surgical intervention is necessary and when the benefit-risk balance is unfavorable.

## Conclusion

Percutaneous pinning is a relatively safe treatment method for unstable distal radius fracture. Significant complications are rare, but the rate of minor complications is high. The application of rigorous definitions and grading systems in further studies on this topic would provide comparable and comprehensive data on complications after DRF CRPP.

## Data Availability

Not applicable.
